# The role of estrogen receptor-beta gene +1730G/A polymorphisms in recurrent pregnancy loss: A protocol for systematic review and meta-analysis

**DOI:** 10.1097/MD.0000000000024398

**Published:** 2021-02-19

**Authors:** Fangxiang Mu, Minge Shi, Li Huang, Dafen Wang, Aiqun Shen

**Affiliations:** a Department of Obstetrics and Gynecology, The First Affiliated Hospital of Chongqing Medical University; b Department of Obstetrics and Gynecology, Chongqing People's Hospital (Tongliang District); c Department of Obstetrics and Gynecology, Chongqing Maternal and Child Health Hospital (Shapingba District); d Department of Obstetrics and Gynecology, Chongqing People's Hospital (Bishan District); e Department of Obstetrics and Gynecology, Tongji Hospital Affiliated to Tongji University, Shanghai, PR China.

**Keywords:** +1730G/A, estrogen receptor-beta, recurrent pregnancy loss, single nucleotide polymorphism

## Abstract

**Objective:**

To identify the role of estrogen receptor-beta (ER-β) gene +1730G/A (rs4986938) polymorphisms in recurrent pregnancy loss (RPL).

**Methods:**

All relevant case-control studies will be systematically searched in multiple databases including PubMed, Embase, Cochrane Library, Web of Science, China National Knowledge Internet (CNKI), Wanfang and Cqvip. Both pooled odds rations (ORs) and 95% confidence intervals (CIs) will be used to assess the association between ER-β gene +1730G/A polymorphisms and RPL risk. The publication bias will be evaluated using Egger test.

**Results:**

ER-β gene +1730G/A variation may be associated with a higher risk of RPL in Caucasian population.

**Conclusions:**

The findings of this meta-analysis will provide high-quality evidence for the association between ER-β gene +1730G/A polymorphisms and RPL, facilitating clinical practice and further scientific studies.

**OSF registration number:**

10.17605/OSF.IO/EW9FB.

## 1 Introduction

Recurrent pregnancy loss (RPL) refers to 2 or more failed pregnancies in clinic and occurs in up to 5% of reproductive age couples.^[1,2]^ It is universally recognized that the occurrence of RPL is associated with diverse factors, such as chromosomal abnormalities, uterine malformation, thrombophilia, infection, endocrine disorders, immune defects, environment and lifestyles.^[3]^ However, approximately 50% of patients with RPL still have no explanations for their pregnancy losses.^[4]^

In recent years, the effect of hormones on the reproduction has been researched extensively, and the alternations of the hypothalamus-pituitary-ovarian (HPO) axis factors are shown a negative impact on the fertility and pregnancy. There is an evidence that the genetic polymorphisms influencing the function of genes which participate in the regulation of HPO axis may be related to RPL.^[5]^ As one of the most important sex hormones in the female reproductive system, the estrogen which acts through estrogen receptors (ER) to exert the physiological effects is found to play a crucial role in the maintenance of pregnancy.^[6]^ ER can be classified into 2 subtypes: ER-α and ER-β, which are respectively coded by ER-α and ER-β genes located on different chromosomes.^[7]^ Although ER-α and ER-β share high homology, they manifest different functions, sometimes even the opposite actions.^[8]^ ER-α performs a crucial function in fertilization, while ER-β in the normal ovulation and regulation of follicular growth.^[9,10]^ Wu et al.^[11]^ found that an apparent conversion from ER-α to ER-β expression was displayed in the myometrium during pregnancy, indicating a potential role of ER-β in normal pregnancy.

ER-β gene located on chromosome 14q22-24 is composed of 8 exons. As one of the common single nucleotide polymorphisms (SNPs) in ER-β gene, the G/A exchange at nucleotide 1730 in the 3’ untranslated region (3’-UTR) of exon 8 (+1730 G/A, rs4986938) is investigated substantially in various gynecological diseases, especially in RPL.^[7,12–14]^ However, it remains unclear about the association between ER-β gene +1730G/A polymorphisms and RPL risk. Therefore, we aim to perform a meta-analysis to further identify the association between ER-β gene +1730G/A polymorphisms and RPL risk based on the currently-available evidence.

## 2 Methods

The data in this study are accessed from openly available datasets, thus there is no need to get the approval from the Institutional Review Board of hospital.

### 2.1 Protocol registration

The prospective registration has been approved by the Open Science Framework (OSF) registries (https://osf.io/ew9fb), and the registration number is 10.17605/OSF.IO/EW9FB. The protocol was written following the Preferred Reporting Items for Systematic Reviews and Meta- Analyses Protocols (PRISMA-P) statement guidelines.^[15]^

### 2.2 Literature search

**Figure 1:**
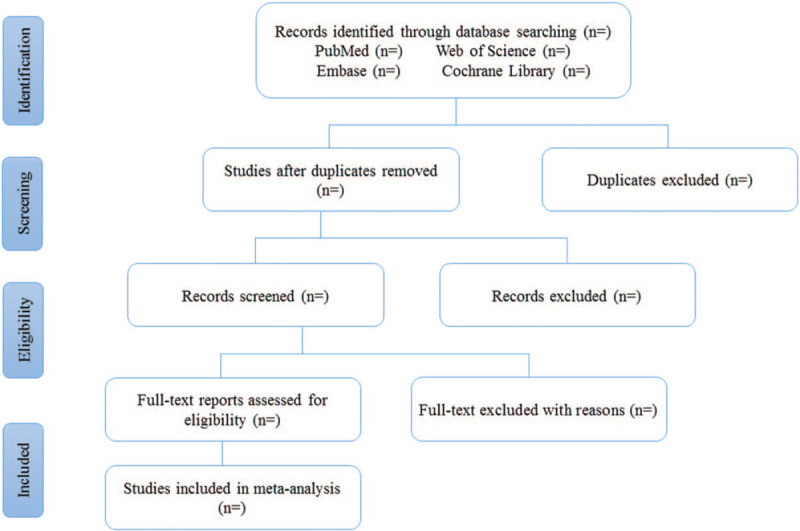
The flow chart of study selection process.

All case-control studies on the association between ER-β gene +1730G/A polymorphisms and RPL risk will be systematically searched in various databases, including PubMed, Embase, Cochrane Library, Web of Science, China National Knowledge Internet (CNKI), Wanfang and Cqvip. The search terms will include “Habitual abortions” OR “Miscarriage, recurrent” OR “Recurrent miscarriage” OR “Recurrent miscarriages” OR “Abortion, recurrent” OR “Recurrent abortion” OR “Recurrent abortions” OR “Recurrent early pregnancy loss” OR “Recurrent pregnancy loss” OR “Recurrent fetal loss” OR “Recurrent pregnancy wastage” OR “Recurrent embryo loss” OR “Recurrent fetal death” OR “Unexplained recurrent spontaneous abortion” OR “URSA” OR “Unexplained habitual abortion” AND “Gene” OR “Polymorphism” OR “Allele” OR “Genotype” OR “Variant” OR “Variation” OR “Mutation” OR “1730 G/A” OR “rs4986938” OR “G1730A” AND “Estrogen receptor” OR “ER” OR “Estrogen receptors” OR “Receptors, estrogen, type II” OR “Estrogen receptor type II” OR “Estrogen receptors type II” OR “Estrogen nuclear receptor” OR “Nuclear receptor, estrogen” OR “Receptor, estrogen nuclear” OR “ER β” (See Table 1 and Fig. 1).

**Table 1:** Search strategy of PubMed.

No.	Search items
#1	(((((((((((((((((“Abortion, Habitual” [Mesh]) OR (Habitual Abortion [Title/Abstract])) OR (Habitual Abortions [Title/Abstract])) OR (Miscarriage, Recurrent [Title/Abstract])) OR (Recurrent Miscarriage [Title/Abstract])) OR (Recurrent Miscarriages [Title/Abstract])) OR (Abortion, Recurrent [Title/Abstract])) OR (Recurrent Abortion [Title/Abstract])) OR (Recurrent Abortions [Title/Abstract])) OR (Recurrent Early Pregnancy Loss [Title/Abstract])) OR (recurrent pregnancy loss [Title/Abstract])) OR (recurrent fetal loss [Title/Abstract])) OR (recurrent pregnancy wastage [Title/Abstract])) OR (recurrent embryo loss [Title/Abstract])) OR (recurrent fetal death [Title/Abstract])) OR (unexplained recurrent spontaneous abortion [Title/Abstract])) OR (URSA [Title/Abstract])) OR (Unexplained habitual abortion [Title/Abstract])
#2	(((((((((((((“Receptors, Estrogen” [Mesh]) OR (Estrogen Receptors [Title/Abstract])) OR (Receptors, Estrogen, Type II [Title/Abstract])) OR (Estrogen Receptor Type II [Title/Abstract])) OR (Estrogen Receptors Type II [Title/Abstract])) OR (Estrogen Nuclear Receptor [Title/Abstract])) OR (Nuclear Receptor, Estrogen [Title/Abstract])) OR (Receptor, Estrogen Nuclear [Title/Abstract])) OR (estrogen receptor [Title/Abstract])) OR (ER [Title/Abstract])) OR (ERβ[Title/Abstract])) OR (G1730A [Title/Abstract])) OR (rs4986938 [Title/Abstract])) OR (1730G/A [Title/Abstract])
#3	#1 AND #2

### 2.3 Inclusion and exclusion criteria

Inclusion criteria:

1. case-control studies;
2. patients diagnosed as RPL in the case group, while those without RPL in the control group;
3. studies that provided the specific genotypes or allele distribution of patients in 2 groups;
4. no statistical significance for the *P* value of Hardy-Weinberg equilibrium (HWE) in control group.

Exclusion criteria:

1. studies unable to extract the effective data;
2. animal experiments, meta-analyses, reviews, letters or editorial articles;
3. studies published not in English.

### 2.4 Data extraction and quality assessment

Two authors will be responsible for extracting the data of publishing articles based on the inclusion and exclusion criteria. The extracted data includes the first author, year of publication, ethnicity, country, genotyping method, quality assessment, genotype distribution and frequency, as well as the value of *HWE* (*p*).

Two authors will be involved in assessing the quality of studies using the modified Newcastle-Ottawa Scale (NOS) which included 3 aspects: patient selection, comparability of study groups and assessment of outcomes. The third author will participate in assessment if there were conflicts. The total score of this scale is 10. The studies with scores <5 and scores ≥5 will be respectively regarded low or moderate quality and high quality.

### 2.5 Statistical analysis

Statistical analysis will be conducted using STATA 14.0 software (Stata Corporation, College Station, TX, USA). The relationship between ER-β gene +1730G/A polymorphisms and RPL risk will be identified by pooled odds rations (ORs) and 95% confidence intervals (CIs). Both *Q* and *I*^2^ tests will be adopted to evaluate the heterogeneity of ORs. No significant heterogeneity will be shown when *Q* > 0.1 and *I*^2^ < 50%. According to the heterogeneity, fixed-effect model (without heterogeneity) and random-effect model (with heterogeneity) will be used, respectively. The publication bias will be assessed using Egger test. *P* < .05 will be regarded as statistically significant.

## 3 Discussion

The maintenance of pregnancy depends on the levels of estrogen and progestogen in the maternal body and their receptor expressions in the cell tissue. A series of biological effects mediated by estrogen as a steroid hormone play an important role in the female reproductive system. The evidence suggests that ER gene polymorphisms can influence diverse estrogen-dependent pathways probably affecting the vascular tone and flow, consequently resulting in disruption of pregnancy maintenance.^[7]^ This estrogen-mediated influence is mainly related to the structural domain of ER-α and ER-β binding to DNA.^[16]^ Although enormous studies have investigated the role of ER gene polymorphisms in RPL,^[17–20]^ there is still short of systemic research.

Approximately 85 SNP sites are reported in the ER-β gene, one of which is the silent G/A polymorphic site located at +1730 G/A of exon 8 that can be cut open by AluI restriction enzyme.^[21]^ Although +1730 G/A polymorphisms do not cause amino acid changes in the ER-β gene, the linkage disequilibrium with various regulatory sequence variations may occur, consequently affecting the gene function or expression.^[22]^ There is an evidence that illustrates the functional effect of 3’-UTR-located SNPs on both gene expression and local RNA structure and regards them as the reason for disease-related SNPs in non-protein-coding transcribed sequences.^[23]^ Meanwhile, the 3’-UTR in most protein-coding genes is also found the objects for microRNAs. In the 3’-UTR sequences of ER-β gene where +1730 G/A is located, however, none of them occurred despite a possible presence of microRNA. Enormous studies conducted in different populations showed no apparent correlation between ER-β gene +1730 G/A polymorphisms and RPL risk.^[12,17,24]^ To gain more information of aspirin in RM, more large-scale RCTs should be conducted further.

Methodology and quality of reporting in meta-analyses are crucial to public health and clinical decision-making. Although numerous studies have been reported, there is no consensus on the association between ER-β gene +1730 G/A variation and RPL risk. In the meta-analysis, we plan to assess the role of ER-β gene +1730 G/A polymorphisms in RPL regarding different genotypes. A variety of databases will be searched to ensure the comprehensiveness of information retrieval.

## Author contributions

AQS, FXM, and MES designed this study. FXM, LH, and DFW collected the data. FXM and MES wrote the manuscript. AQS critically reviewed, edited and approved the manuscript. All authors read and approved the final manuscript.

**Conceptualization:** Fangxiang Mu, Minge Shi, Aiqun Shen.

**Data curation:** Fangxiang Mu, Li Huang, Dafen Wang.

**Writing – original draft:** Fangxiang Mu, Minge Shi.

**Writing – review & editing:** Aiqun Shen.
